# The Women’s Microbiome: Molecular Insights, Clinical Gaps, and Future Frontiers in Precision Health with Implications for Gulf Cooperation Council Populations

**DOI:** 10.3390/ijms27062521

**Published:** 2026-03-10

**Authors:** Muriel Tahtouh Zaatar, Rima Othman, Mohammed Abushawish, Michel Akl, Mohamad Taha Alachkar, Ghaya Almatboona, Fatma Alriyami, Aljoud Alshaibani, Dana Ashkanani, Munira Basharova, Mohammad Imam, Nadia Khassay, Mila Souha Mikhael, Rozhin Naderi Far, Sophia Shaqra, Kiara Verwey, Alika Suleimanova, Mariam Yousafzada, Yuliya Burmagina

**Affiliations:** 1 Department of Biological and Physical Sciences, American University in Dubai, Dubai P.O. Box 28282, United Arab Emirates; mohammed.abushawish@mymail.aud.edu (M.A.); michel.akl@mymail.aud.edu (M.A.); mohamadtaha.alachkar@mymail.aud.edu (M.T.A.); ghaya.almatboona@mymail.aud.edu (G.A.); fatma.alriyami@mymail.aud.edu (F.A.); aljoud.alshaibani@mymail.aud.edu (A.A.); dana.ashkanani@mymail.aud.edu (D.A.); munira.basharova@mymail.aud.edu (M.B.); mohammad.imam@mymail.aud.edu (M.I.); nadia.khassay@mymail.aud.edu (N.K.); mila.mikhael@mymail.aud.edu (M.S.M.); rozhin.naderifar@mymail.aud.edu (R.N.F.); sophia.shaqra@mymail.aud.edu (S.S.); kiara.verwey@mymail.aud.edu (K.V.); alika.suleimanova@mymail.aud.edu (A.S.); maryam.yosofzadah@mymail.aud.edu (M.Y.); 2 Department of Pulmonary Medicine and Critical Care, Johns Hopkins University, Baltimore, MD 21205, USA; 3 Mediclinic City Hospital, Dubai P.O. Box 505030, United Arab Emirates; juliamikhael@yahoo.com; 4 Optima Medical Center, Dubai P.O. Box 554450, United Arab Emirates

**Keywords:** women’s microbiome, gut–vaginal axis, estrobolome, precision medicine, probiotics, pregnancy, fertility, microbiome–hormone interactions, artificial intelligence, women’s health

## Abstract

The human microbiome has emerged as a central regulator of health and disease; however, women-specific microbiome research has only recently gained focused scientific attention. Accumulating evidence demonstrates that microbial ecosystems across the gut, vagina, skin, breast tissue, and reproductive tract are dynamically shaped by female hormones, life-stage transitions, and environmental exposures. These interactions influence immune regulation, metabolic homeostasis, reproductive outcomes, mental health, and cancer risk, in part through microbiome-mediated endocrine pathways such as the estrobolome. Advances in high-resolution molecular technologies—including metagenomics, metabolomics, spatial and single-cell profiling, and artificial intelligence-driven modeling—have shifted microbiome research from descriptive taxonomy toward functional, mechanistic, and predictive science. These approaches highlight microbial function and metabolite production as stronger determinants of health outcomes than taxonomic composition alone. Nonetheless, major gaps persist, including limited causal evidence, methodological heterogeneity, underrepresentation of non-Western populations, and barriers to clinical translation. Microbiome-targeted interventions, including probiotics, prebiotics, postbiotics, and emerging microbiota-based therapies, have garnered increasing interest in women’s health. Select *Lactobacillus* and *Bifidobacterium* strains show potential in modulating vaginal and gastrointestinal health, pregnancy outcomes, and immune function; however, clinical effects remain highly strain-specific and context-dependent. Discrepancies between experimental findings, commercial claims, and validated clinical use underscore the need for rigorous, women-centered trials and standardized outcome measures. This narrative review synthesizes current molecular insights into the women’s microbiome across endocrine interactions, pregnancy, reproductive and metabolic health, lifestyle influences, and microbiome-based therapeutic strategies. We integrate clinical perspectives to identify diagnostic and translational challenges and propose future directions emphasizing precision microbiome medicine, validated biomarkers, careful evaluation of microbiome-targeted interventions, and inclusive research frameworks, including populations from the Gulf Cooperation Council (GCC). Collectively, this review positions the microbiome as a critical yet underutilized axis in women’s health and outlines a roadmap toward personalized, evidence-based care across the female lifespan.

## 1. Introduction

Scientific interest in the human microbiome has expanded rapidly over the past two decades, transforming understanding of microbial communities from passive colonizers to integral regulators of immunity, metabolism, and disease susceptibility. Although early observations of human-associated microorganisms date back to the seventeenth century, major advances in microbiome research were enabled by the advent of high-throughput molecular sequencing technologies in the early 2000s [1]. By 2025, women’s microbiome research has emerged as a distinct and increasingly important field, driven by growing recognition that sex-specific biological factors profoundly shape microbial composition, function, and host interactions across multiple body sites.

Large-scale population studies have demonstrated that microbial communities vary significantly according to age, sex, ethnicity, and hormonal status, underscoring the limitations of extrapolating findings from male-dominated or mixed cohorts to women’s health outcomes [2]. These observations highlight the necessity for research approaches specifically designed to capture female physiological dynamics across the lifespan. Accordingly, the women’s microbiome has become central to advancing precision medicine strategies aimed at improving diagnostic accuracy, disease risk stratification, and therapeutic outcomes.

A major driver of this field is the recognition that female sex hormones act as ecological regulators of microbial ecosystems. Fluctuations in estrogen and progesterone across the menstrual cycle, pregnancy, and menopause influence microbial stability and community structure within the gut, vaginal, and urinary tract microbiomes, determining whether protective or dysbiotic microbial states prevail [3]. Estrogen represents a critical biological interface linking microbial activity with immune regulation and reproductive physiology. Conversely, microbial communities actively modulate hormone metabolism through enzymatic pathways, forming a bidirectional hormone–microbiome axis with important implications for women’s health [4]. Disruptions to this axis have been associated with adverse reproductive outcomes, including infertility, altered reproductive tract function, and hormone-sensitive disease states [5].

Despite methodological progress, significant population-level disparities remain within the literature. Women from non-Western regions continue to be underrepresented in microbiome studies, limiting the generalizability of current findings. This gap is particularly relevant in the Gulf Cooperation Council (GCC) region, where emerging metagenomic analyses have identified distinct gut microbiome signatures associated with insulin resistance, adiposity, and metabolic dysfunction among Saudi women [6]. While this gap has important biological and translational implications. GCC populations are characterized by distinct dietary patterns, high prevalence of obesity, insulin resistance, and type 2 diabetes, widespread antibiotic exposure, and unique reproductive and obstetric profiles [6]. Each of these factors is known to independently shape gut and vaginal microbiome composition, microbial metabolite production, and estrogen metabolism. Consequently, microbiome–host interactions described in predominantly Western cohorts may not be directly generalizable to women in this region. Addressing such regional differences is essential for developing inclusive, culturally relevant, and globally applicable microbiome-based interventions.

Concurrently, women’s microbiome research has undergone a methodological transition from descriptive taxonomy toward functional and mechanistic investigation. While early studies relied heavily on 16S rRNA gene sequencing, recent work increasingly employs shotgun metagenomics, metabolomics, and transcriptomics to achieve strain-level resolution and functional characterization. These approaches have demonstrated that microbial metabolites and enzymatic activity, rather than taxonomic composition alone, often provide stronger predictive value for health outcomes, challenging traditional assumptions regarding microbial “benefit” or “pathogenicity” [7,8,9]. Advances in spatial transcriptomics have further revealed localized host–microbe interactions within reproductive tissues, offering new insights into implantation failure, immune tolerance, and reproductive dysfunction [10].

Within this evolving framework, natural products have emerged as a critical yet underexplored interface between environmental exposure and host–microbiome interactions in women’s health. Dietary components, plant-derived bioactive compounds, fermented foods, probiotics, and postbiotics exert measurable effects on microbial composition and function across both the gut and vaginal ecosystems. These interventions influence key molecular pathways, including estrogen metabolism, immune signaling, epithelial barrier integrity, and microbial metabolite production, thereby shaping systemic, metabolic, and reproductive health outcomes.

In the gut, diet-derived fibers and polyphenols modulate the activity of the estrobolome, the collection of microbial genes involved in estrogen metabolism, through regulation of enzymes such as β-glucuronidase and sulfatase [11,12,13]. Alterations in this pathway influence circulating estrogen levels and have been implicated in hormone-sensitive conditions, including metabolic dysfunction, breast cancer risk, and menopausal symptomatology. In parallel, microbial fermentation of natural substrates generates short-chain fatty acids, indole derivatives, and secondary bile acids that act as signaling molecules linking the microbiome to immune modulation and neuroendocrine pathways [14].

Within the vaginal microbiome, natural product–based interventions, including probiotic formulations and diet-mediated metabolic effects, influence lactic acid production, vaginal pH, epithelial tight junction integrity, and immune defense mechanisms. Lactobacillus-dominated communities generate D- and L-lactic acid isomers that maintain an acidic microenvironment and suppress pathogenic overgrowth, while dysbiosis is associated with accumulation of pro-inflammatory metabolites such as succinate [15,16,17,18]. These molecular shifts are strongly associated with bacterial vaginosis, susceptibility to infection, infertility, and adverse pregnancy outcomes.

Together, these foundational concepts establish the women’s microbiome as a dynamic, hormone-responsive system shaped by molecular interactions between microbial metabolism, host physiology, and environmental exposures including natural products. Building upon this framework, the following sections examine site-specific microbiomes, reproductive and metabolic health outcomes, lifestyle influences, and emerging therapeutic strategies, while highlighting persistent gaps that must be addressed to translate molecular insights into equitable and effective clinical practice (Table 1).

## 2. The Gut Microbiome and Women’s Health

### 2.1. Gut Microbiome and Female Endocrine Function

The gut microbiome plays a central role in regulating female endocrine health through its influence on estrogen, insulin, and androgen homeostasis across the lifespan. A key mediator of this interaction is the estrobolome, defined as the subset of gut microbial genes involved in estrogen metabolism and enterohepatic recirculation [19,20]. Estrogen conjugates excreted in bile can be deconjugated by microbial β-glucuronidase enzymes, allowing biologically active estrogens to re-enter systemic circulation and modulate reproductive, metabolic, and immune processes [20,21].

This gut–endocrine axis is particularly relevant during hormonally dynamic periods such as puberty, when microbial activity may influence reproductive maturation and endocrine regulation [21]. Disruption of gut microbial balance can impair estrogen recycling, leading to altered hormonal availability and downstream metabolic and reproductive consequences. Gut dysbiosis has been consistently associated with polycystic ovary syndrome (PCOS), obesity, insulin resistance, and metabolic syndrome conditions characterized by overlapping endocrine and inflammatory disturbances [22,23]. Women with PCOS exhibit reduced gut microbial diversity, altered short-chain fatty acid production, and changes in estrobolome-associated enzymatic activity, suggesting functional links between microbial metabolism and endocrine dysfunction [21,23].

**Figure 1:**
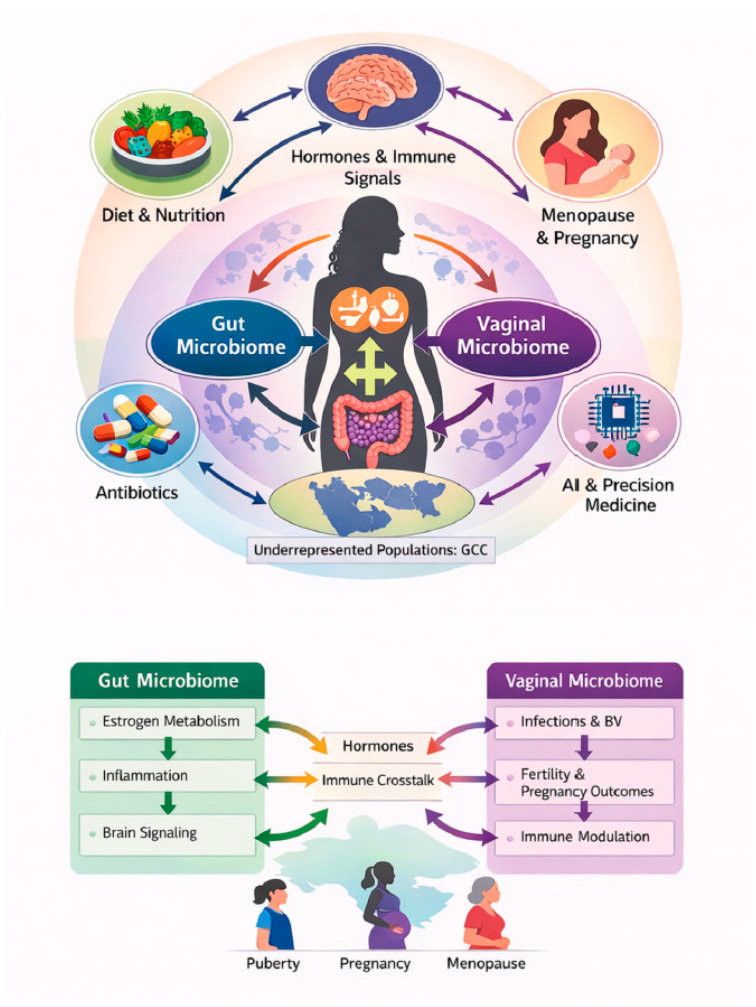
Natural products modulate microbiome–host interactions across the female lifespan. This schematic illustrates bidirectional interactions between the gut and vaginal microbiomes and their modulation by hormonal, immune, metabolic, and environmental factors throughout key female life stages, including puberty, pregnancy, and menopause. The gut microbiome influences systemic estrogen metabolism (estrobolome activity), inflammatory tone, and gut–brain signaling, while the vaginal microbiome regulates epithelial barrier integrity, susceptibility to infection (e.g., bacterial vaginosis), fertility, pregnancy outcomes, and local immune modulation. Hormonal and immune signaling act as central integrative pathways linking these microbial ecosystems. External modifiers such as diet and nutrition, antibiotic exposure, and emerging precision medicine approaches, including artificial intelligence–guided microbiome profiling, shape microbial composition and function. Underrepresented populations, particularly women from GCC countries, are highlighted to emphasize regional variability in microbiome–host interactions and the need for population-specific mechanistic and translational research. Arrows indicate dynamic, bidirectional interactions rather than direct causality.

Across the lifespan, gut microbiome composition continues to evolve in response to hormonal changes. Postmenopausal women often exhibit increased microbial diversity as ovarian estrogen production declines; however, estrogen metabolism remains active through β-glucuronidase-producing taxa such as *Ruminococcaceae* and *Clostridia*, which continue to influence systemic estrogen levels [24]. Beyond reproductive health, dysregulated estrobolome activity has been implicated in estrogen-dependent malignancies. Increased microbial estrogen reactivation has been proposed as a contributor to breast cancer risk, although existing evidence remains largely associative and causality has not been established [14,25]. Regional factors prevalent in GCC populations, including metabolic disease burden and repeated antibiotic exposure, are likely to alter estrobolome composition and β-glucuronidase activity, potentially modifying estrogen recycling and systemic hormone availability. These differences may influence susceptibility to estrogen-sensitive conditions and responsiveness to microbiome-targeted interventions. Collectively, these findings highlight the gut microbiome–endocrine axis as a critical determinant of women’s health, while underscoring the need for longitudinal and functional studies to clarify mechanistic pathways (Figure 1).

### 2.2. Gut Microbiome, Pregnancy, and the Maternal–Fetal Axis

Pregnancy is characterized by profound metabolic, immunological, and vascular adaptations that are paralleled by dynamic shifts in the maternal gut microbiome. These microbial changes influence maternal insulin sensitivity, immune tolerance, and inflammatory signaling, thereby shaping pregnancy outcomes [26,27]. Alterations in gut microbial composition and metabolite production have been associated with gestational diabetes mellitus, pre-eclampsia, and preterm birth [26,28].

The maternal gut microbiome plays a key role in regulating the maternal–fetal axis through microbially derived metabolites that influence immune activation, placental vascular function, and blood pressure regulation [29]. Emerging evidence suggests that maternal microbiota may be transmitted to the offspring during and after birth, contributing to fetal immune development and long-term metabolic programming [27,28]. These observations position pregnancy as a critical window during which microbiome composition may exert lasting intergenerational effects.

Dietary factors are important modulators of the maternal gut microbiome. High-fiber diets and probiotic supplementation have been proposed as strategies to promote beneficial microbial profiles and anti-inflammatory metabolite production during pregnancy [26,27]. However, intervention studies have yielded inconsistent results, reflecting heterogeneity in probiotic strains, timing of exposure, and outcome measures [26,30]. Notably, pregnancy-related microbiome dynamics may be particularly relevant in GCC populations, where higher rates of gestational diabetes, cesarean delivery, and antibiotic exposure are reported. These factors are known to influence maternal gut and vaginal microbiome stability, microbial metabolite signaling, and immune adaptation at the maternal–fetal interface. The lack of longitudinal microbiome data from pregnant women in the GCC therefore represents a critical gap in understanding region-specific risks and opportunities for intervention [30,31]. These gaps underscore the need for region-specific longitudinal studies and culturally tailored interventions.

### 2.3. Gut–Brain–Hormone Axis in Women

The gut–brain–hormone axis provides a mechanistic framework for understanding how microbiome function interfaces with neuroendocrine signaling to influence mental health in women. Gut microbes regulate neurotransmitter availability, immune activation, and hypothalamic–pituitary–adrenal axis activity, thereby modulating mood and stress responsiveness [17,32]. In particular, microbial effects on serotonin metabolism and estrogen signaling represent key biological interfaces linking gut microbial activity to hormonally sensitive neurobehavioral states. Although associations between gut microbiome alterations and mood disorders are increasingly reported, causal molecular mechanisms remain incompletely defined. Observational studies and Mendelian randomization analyses suggest a potential contribution of gut microbiota to postpartum depression; however, direct mechanistic evidence linking specific taxa, metabolites, or signaling pathways to clinical phenotypes is limited [33,34,35]. Pregnancy-associated shifts in microbial composition may alter neuroimmune and endocrine signaling in susceptible individuals, but the functional mediators of these effects remain to be elucidated. Microbiome-targeted strategies, including probiotic supplementation, have been proposed as potential modulators of hormone-associated mood vulnerability. To date, clinical evidence remains insufficient, and strain-specific effects have not been systematically evaluated [32]. Moreover, population-specific modifiers such as diet, lifestyle, and environmental exposures may further shape gut–brain–hormone interactions, particularly in underrepresented regions such as the GCC. Collectively, these findings underscore the promise of the gut–brain–hormone axis as a conceptual framework while highlighting the need for molecularly resolved studies and rigorously designed clinical trials to enable translational application.

### 2.4. Molecular Pathways Underpinning Microbiome–Host Crosstalk

At the molecular level, microbiome–host interactions are mediated by microbial enzymes, metabolites, and host receptors that collectively regulate endocrine, immune, and neural signaling. In the gut, microbial β-glucuronidase activity governs estrogen deconjugation and enterohepatic recirculation, directly influencing systemic estrogen availability and receptor signaling. Dysregulation of this pathway alters inflammatory tone, adiposity, and cancer risk. Additional gut-derived metabolites, including short-chain fatty acids and indole compounds, modulate immune cell differentiation, epithelial integrity, and blood–brain barrier signaling, providing mechanistic links between microbial activity and neuroimmune regulation.

Within the vaginal microbiome, host protection is mediated primarily through metabolite-driven modulation of the local microenvironment. Lactobacillus-dominated communities produce lactic acid isomers that maintain acidic pH, reinforce epithelial tight junctions, and suppress pathogenic overgrowth. Disruption of this metabolic balance leads to accumulation of succinate and other pro-inflammatory metabolites, promoting immune dysregulation and epithelial vulnerability. These molecular shifts are strongly associated with bacterial vaginosis, infertility, and adverse pregnancy outcomes.

**Figure 2:**
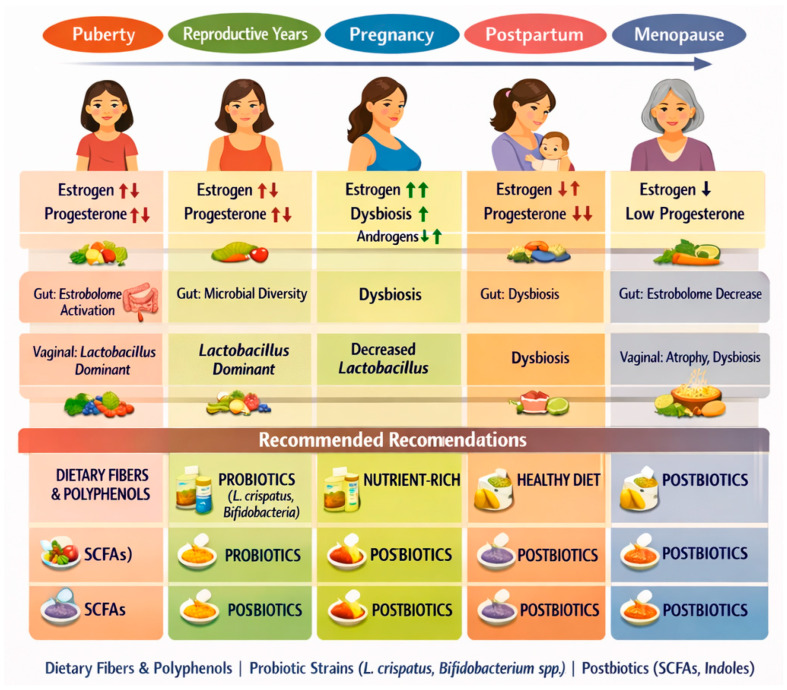
Microbiome dynamics across the female lifespan and stage-specific microbiome-targeted recommendations. Illustration depicting hormonal fluctuations across key female life stages—puberty, reproductive years, pregnancy, postpartum, and menopause—and their associated effects on gut and vaginal microbiome composition and function. The upper panel shows hormonal fluctuations over the female lifespan with consequent changes in gut and vaginal microbiomes. The lower panel highlights recommended microbiome-supportive interventions, including dietary fibers and polyphenols, probiotics (e.g., *Lactobacillus crispatus*, *Bifidobacterium* spp.), nutrient-rich or healthy dietary patterns, and postbiotics such as short-chain fatty acids (SCFAs) and indole derivatives.

Hormonal fluctuations across puberty, pregnancy, and menopause dynamically reshape these molecular interactions. Estrogen regulates glycogen availability in the vaginal epithelium, altering microbial substrate utilization, while systemic inflammation feeds back to influence microbial composition and function. Natural product-derived interventions intersect with these pathways by modifying substrate availability, enzymatic activity, and metabolite signaling, highlighting their potential to restore microbiome-mediated homeostasis at the molecular level. Together, these findings support an integrated gut–vaginal–endocrine framework in which microbial, hormonal, and immune interactions dynamically shape women’s health across the lifespan (Figure 2).

## 3. The Vaginal Microbiome

### 3.1. Vaginal Microbiome Basics and Molecular Signaling

The vaginal microbiome is a critical determinant of female reproductive tract health, regulating local pH, epithelial barrier integrity, and immune signaling through metabolite-mediated mechanisms. In most healthy reproductive-age women, the vaginal microbiota is dominated by *Lactobacillus* species, which maintain an acidic environment primarily via lactic acid production [36]. This low-pH milieu restricts pathogen growth and supports mucosal defense.

Beyond pH regulation, *Lactobacillus*-derived metabolites exert direct effects on epithelial and immune function. Lactic acid enhances transepithelial resistance and upregulates tight junction protein expression in cervicovaginal epithelial cells, indicating a direct role in strengthening mucosal barrier integrity [37]. At the transcriptional and epigenomic level, *Lactobacillus crispatus* promotes epithelial homeostasis by suppressing inflammatory signaling and modulating chromatin accessibility, whereas dysbiosis-associated species such as *Gardnerella vaginalis* induce epithelial stress responses and innate immune activation [38].

Metabolite signaling further shapes local immune tone. Exposure to lactic acid under acidic conditions characteristic of *Lactobacillus*-dominant communities dampens pro-inflammatory cytokine responses, while metabolites such as succinate and short-chain fatty acids associated with dysbiotic states fail to confer similar protection and may promote inflammation with sustained exposure [39]. Multi-omics studies demonstrate that metabolite profiles are stronger predictors of vaginal pH, inflammation, and epithelial health than microbial taxonomy alone [40]. Importantly, individual *Lactobacillus* species generate distinct metabolic signatures, contributing in species-specific ways to epithelial and immunological homeostasis [41].

Recent genomic analyses have revealed extensive gene-content diversity across vaginal microbial taxa, reinforcing the concept that vaginal health is governed by functional capacity and metabolite output rather than the mere presence of *Lactobacillus* [42]. Despite these advances, the molecular mechanisms through which specific species and metabolites influence host signaling remain incompletely defined, representing a key gap in vaginal microbiome research.

### 3.2. Dysbiosis and Recurrent Infections

Vaginal dysbiosis is characterized by depletion of *Lactobacillus* species and overgrowth of diverse anaerobic bacteria, resulting in elevated pH, impaired epithelial defenses, and increased susceptibility to infection. Dysbiotic vaginal states have been associated with bacterial vaginosis, vulvovaginal candidiasis, urinary tract infections, and upper reproductive tract disorders [43,44]. Recurrence is common, reflecting limited microbial resilience and incomplete restoration of protective communities following treatment.

Antibiotic therapy remains the primary treatment for many vaginal infections; however, frequent or inappropriate antibiotic use contributes to microbial instability, recurrence, and antimicrobial resistance [45]. While antibiotics may transiently reduce pathogen burden, they often disrupt beneficial *Lactobacillus* populations, perpetuating cycles of dysbiosis. Increasing evidence suggests that vaginal microbial resilience is shaped by host factors, microbial interactions, and crosstalk with the gut microbiome, although these relationships remain poorly characterized [46].

Probiotic interventions, particularly those containing *Lactobacillus* strains, have been investigated as adjunctive therapies for recurrent vaginal infections. Although some studies report modest benefit, outcomes remain inconsistent, especially in polymicrobial dysbiosis where single-strain approaches may be insufficient [47]. Collectively, these findings underscore the need for microbiome-guided therapies that target species-specific functions and metabolic pathways rather than relying solely on broad-spectrum antimicrobial strategies.

### 3.3. Vaginal Microbiome and Sexually Transmitted Infections

The vaginal microbiome is increasingly recognized as a determinant of susceptibility to sexually transmitted infections (STIs) through its effects on mucosal immunity, epithelial integrity, and inflammatory signaling. *Lactobacillus*-dominant communities are associated with reduced STI risk, whereas diverse anaerobic microbiota resembling bacterial vaginosis confer increased vulnerability [48].

Longitudinal cohort studies demonstrate that *Lactobacillus*-deficient cervicovaginal communities are associated with a significantly increased risk of HIV acquisition, potentially mediated by elevated inflammatory cytokines and recruitment of activated immune target cells [49]. Meta-analyses support an overall association between vaginal dysbiosis and HIV susceptibility, though effect sizes vary across populations and mechanistic pathways remain incompletely defined [50].

In the context of human papillomavirus infection, longitudinal and systematic studies indicate that *Lactobacillus*-dominant microbiota, particularly communities enriched in *Lactobacillus gasseri*, are associated with viral clearance, whereas non-*Lactobacillus*-dominant states correlate with persistent high-risk HPV infection and cervical dysplasia [51,52]. Emerging evidence also suggests organism-specific interactions between the vaginal microbiome and STIs such as *Chlamydia trachomatis* and *Trichomonas vaginalis*, highlighting the role of microbial context in pathogen persistence and disease progression [52].

Despite these insights, much of the literature remains cross-sectional and methodologically heterogeneous, with limited control for confounders such as sexual behavior, contraceptive use, and regional variability. Molecular investigations of epithelial barrier disruption, metabolite signaling, and immune modulation in STI susceptibility are particularly scarce in underrepresented regions, including the Gulf Cooperation Council countries. Addressing these gaps will require longitudinal, multi-omic cohorts and microbiome-targeted interventional studies tailored to diverse female populations.

## 4. Microbiome and Reproductive Health

### 4.1. Microbiome and Fertility Outcomes

Accumulating evidence indicates that the female microbiome plays a meaningful role in reproductive success, particularly in the context of assisted reproductive technologies (ART) such as in vitro fertilization (IVF). The vaginal, endometrial, and gut microbiomes have each been implicated in embryo implantation, endometrial receptivity, and early pregnancy maintenance. Among these, a Lactobacillus-dominant microbiome within the female genital tract has most consistently been associated with favorable fertility outcomes [53].

Multiple clinical studies and systematic reviews report higher implantation and clinical pregnancy rates following IVF and intracytoplasmic sperm injection among women with Lactobacillus-dominant vaginal microbiota compared with those exhibiting non-Lactobacillus-dominant or dysbiotic profiles [53,54,55]. The protective effects of Lactobacillus species are attributed to lactic acid-mediated acidification, suppression of pathogenic bacteria, and attenuation of local inflammatory signaling, all of which support epithelial integrity and endometrial receptivity [53].

Conversely, vaginal dysbiosis characterized by increased abundance of anaerobic genera such as Gardnerella and Prevotella has been associated with implantation failure and reduced ART success [54]. Prospective cohort studies suggest that non-Lactobacillus-dominant cervicovaginal microbiota may predict pregnancy failure following embryo transfer, raising interest in microbial profiling as a potential adjunct in fertility care [54]. Emerging evidence further implicates gut microbiome dysbiosis in recurrent spontaneous abortion, highlighting the systemic nature of microbiome–reproductive interactions beyond the reproductive tract alone [56].

Despite these associations, important methodological limitations persist. Variability in sampling techniques, low microbial biomass within the endometrium, and challenges in distinguishing resident uterine microbiota from transcervical contamination complicate interpretation of findings [53,55]. In addition, strain-level heterogeneity within Lactobacillus species appears to influence reproductive outcomes, yet underlying mechanisms remain poorly defined [54]. Although experimental approaches such as antibiotic-free vaginal microbiota transplantation have shown promise in small proof-of-concept studies, robust randomized controlled trials are required before clinical implementation [57]. Overall, advancing microbiome-guided fertility care will require standardized definitions of dysbiosis, mechanistic validation, and interventional evidence.

### 4.2. Microbiome in Polycystic Ovary Syndrome and Endometriosis

PCOS and endometriosis are prevalent reproductive disorders characterized by complex interactions among endocrine dysregulation, immune activation, and chronic inflammation. Increasing attention has focused on the gut microbiome as a potential regulator of these processes through metabolic, hormonal, and immunological pathways [58]. Although dysbiosis has been consistently reported in women with PCOS and endometriosis, causal relationships remain unresolved.

A central mechanistic link involves the estrobolome, the subset of gut microbial genes involved in estrogen metabolism. Gut bacteria capable of producing β-glucuronidase enzymes, including taxa within *Clostridium*, *Bacteroides*, and *Escherichia*, facilitate estrogen deconjugation and enterohepatic recirculation, thereby increasing circulating estrogen availability [29,59]. Excessive β-glucuronidase activity may contribute to estrogen dominance, progesterone resistance, and impaired endometrial receptivity; features commonly observed in both PCOS and endometriosis [60].

Beyond hormonal effects, gut dysbiosis has been linked to immune dysregulation and systemic inflammation. Altered microbial composition may increase intestinal permeability and endotoxin translocation, promoting chronic activation of pro-inflammatory pathways involving cytokines such as interleukin-6 and tumor necrosis factor-α, which have been implicated in ovarian dysfunction and ectopic endometrial lesion progression [61]. Experimental studies suggest that probiotic organisms can modulate immune responses and reinforce epithelial barrier function; however, clinical outcomes in PCOS and endometriosis remain inconsistent and strain-dependent [62].

Current evidence is largely observational and limited by small sample sizes, heterogeneous diagnostic criteria, and inadequate control for confounders such as diet, body mass index, and medication use [58,60]. While integrative multi-omics approaches combining microbiome profiling with hormonal and immune phenotyping have been proposed to identify novel biomarkers, their clinical utility has yet to be established. Consequently, longitudinal and interventional studies are required to determine whether microbiome alterations represent causal drivers or secondary consequences of reproductive disease, particularly in underrepresented populations.

## 5. Microbiome, Lifestyle, and Women’s Wellbeing

### 5.1. Nutrition, Exercise, and Hormonal Cycles

Lifestyle factors, particularly diet and physical activity, exert significant influence on the gut microbiome and interact bidirectionally with female hormonal cycles. Dietary composition shapes microbial diversity, metabolite production, and estrogen metabolism, thereby modulating endocrine homeostasis across the menstrual cycle [63,64]. Fluctuations in estrogen and progesterone during the follicular and luteal phases have been associated with corresponding shifts in gut microbial composition, suggesting dynamic hormone–microbiome feedback mechanisms [63].

Dietary patterns rich in fiber promote the production of short-chain fatty acids, which influence insulin sensitivity, immune regulation, and estrogen signaling. In contrast, high-fat and low-fiber diets have been linked to reduced microbial diversity and dysregulated estrogen metabolism, potentially exacerbating premenstrual symptoms and metabolic disturbances [64,65]. Physical activity further modulates gut microbial composition, with regular exercise associated with increased microbial diversity and enrichment of metabolically beneficial taxa, although sex-specific effects remain insufficiently characterized [66].

Despite growing recognition of these interactions, most studies examining lifestyle-microbiome–hormone relationships in women remain cross-sectional or short-term. Longitudinal investigations incorporating repeated sampling across menstrual cycles, reproductive stages, and lifestyle transitions are scarce, limiting causal inference and the development of personalized lifestyle interventions [63,66].

### 5.2. Skin Microbiome and Women’s Dermatological Conditions

The skin microbiome constitutes a dynamic ecosystem that contributes to barrier integrity, immune balance, and cutaneous homeostasis. In women, hormonal fluctuations across the menstrual cycle, pregnancy, and hormonal contraceptive use alter skin physiology by modulating sebum production, lipid composition, hydration, and pH, thereby reshaping microbial communities [67,68]. These hormonal influences are reflected in measurable shifts in microbial diversity and species composition.

Acne vulgaris is increasingly recognized as a disorder of microbial imbalance rather than simple overgrowth of *Cutibacterium acnes*. Disease severity correlates with reduced microbial diversity and shifts in *C. acnes* phylotypes that promote inflammatory signaling [69]. Similarly, eczema has been associated with diminished microbial heterogeneity and episodic dominance of *Staphylococcus aureus*, contributing to chronic inflammation and epidermal dysfunction [68].

Pregnancy introduces additional physiological complexity. Changes in androgen levels and immune tolerance during later trimesters may exacerbate acne and inflammatory dermatoses, while pregnancy-associated shifts in skin microbial composition have been observed at both taxonomic and functional levels [70,71]. However, direct correlations between microbial changes and dermatological outcomes during pregnancy remain limited.

Everyday exposures, including cosmetics and hormonal contraceptives, further interact with skin microbial communities by altering surface lipids and introducing bioactive compounds. Although the market for “microbiome-friendly” skincare products has expanded rapidly, most commercial claims lack validation in controlled clinical trials [72]. Overall, the hormone–microbiome–skin axis represents a promising but underdeveloped area, underscoring the need for longitudinal, mechanistic studies to guide evidence-based dermatological interventions.

### 5.3. Breast Microbiome and Cancer Risk

The breast tissue microbiome has emerged as a potential modulator of mammary immune homeostasis and carcinogenesis yet remains among the least studied microbial niches in women’s health. Sequencing studies have identified distinct microbial communities in malignant versus normal breast tissue, with tumors enriched in taxa associated with inflammation, including *Escherichia coli* and *Fusobacterium nucleatum*, while healthy tissue is more abundant in potentially protective genera such as *Lactobacillus* and *Sphingomonas* [73,74,75].

Mechanistic evidence suggests that intratumoral microbes may influence cancer progression through immune modulation and genotoxic pathways. Certain *E. coli* strains harbor the pks genomic island encoding the genotoxin colibactin, which induces DNA interstrand cross-links and genomic instability [76,77]. Experimental studies further demonstrate that *F. nucleatum* promotes breast cancer cell migration and immune evasion via microRNA-mediated pathways that impair antigen presentation and cytotoxic immune responses [78,79,80].

Microbial metabolites also shape the tumor microenvironment. β-glucuronidase–producing bacteria may contribute to estrogen reactivation via the estrobolome, potentially increasing hormone-dependent cancer risk, whereas short-chain fatty acids have been associated with enhanced cytotoxic T-cell activity and anti-tumor immunity [81,82,83]. Activation of innate immune pathways, including Toll-like receptor 4 signaling, may further reinforce pro-inflammatory, pro-tumor conditions [84].

Despite these advances, human studies of the breast microbiome are limited by small sample sizes, cross-sectional designs, and variability in contamination controls. Ethnic and geographic diversity remains minimal, and causal relationships are largely unresolved. Consequently, the breast microbiome represents a nascent research frontier requiring longitudinal, prospectively sampled cohorts and integrated multi-omic approaches to establish clinical relevance and translational potential [85].

## 6. Microbiome Interventions: Current and Future Directions

### 6.1. Probiotics, Prebiotics, and Postbiotics for Women

Microbiome-targeted interventions—including probiotics, prebiotics, and postbiotics—have gained substantial attention in women’s health. Although numerous commercial products claim benefits for digestive, reproductive, and hormonal balance, the scientific evidence supporting these claims remains highly variable. A growing body of research underscores that probiotic effects are strain-specific, disease-specific, and context-dependent, highlighting the limitations of generalized formulations marketed toward women [86,87,88].

**Figure 3:**
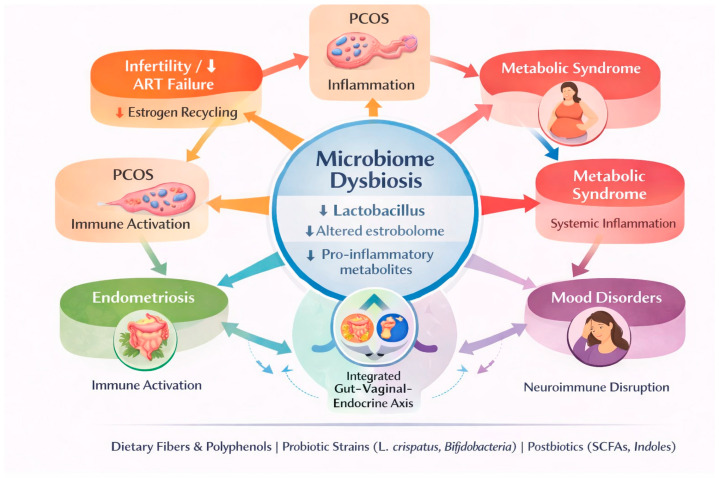
Microbiome dysbiosis as a shared mechanistic hub linking women’s reproductive, metabolic, and neuroimmune disorders. This schematic illustrates how microbiome dysbiosis, characterized by reduced *Lactobacillus* abundance, altered estrobolome activity, and increased production of pro-inflammatory microbial metabolites, functions as a central, integrative driver connecting multiple women’s health conditions. Disrupted estrogen recycling and immune activation are linked to infertility and adverse assisted reproductive technology (ART) outcomes, as well as PCOS. Systemic inflammation and metabolic dysregulation connect dysbiosis to metabolic syndrome, while epithelial barrier dysfunction contributes to recurrent infections. Neuroimmune disruption provides a mechanistic link between microbial imbalance and mood disorders. Arrows indicate interconnected and bidirectional biological pathways rather than direct causality. Dietary fibers and polyphenols, targeted probiotic strains (e.g., *Lactobacillus crispatus*, *Bifidobacterium* spp.), and postbiotics (e.g., short-chain fatty acids and indole derivatives) are highlighted as modulators of microbial function with potential therapeutic relevance.

Clinical studies indicate that select *Lactobacillus* strains, particularly *Lactobacillus crispatus*, can promote vaginal microbiome stability and reduce recurrence of bacterial vaginosis when administered vaginally rather than orally [89,90]. In gastrointestinal contexts, strains such as *Bifidobacterium animalis* and *Lactobacillus rhamnosus* have demonstrated benefits for bowel regularity and gastrointestinal symptoms, while postbiotics, defined bioactive microbial metabolites, may offer more stable symptom control during menopause [91,92,93]. Importantly, these effects cannot be extrapolated across strains or clinical indications (Figure 3).

Despite encouraging findings, substantial gaps persist between scientific evidence and commercial practice. Many probiotic products fail to disclose strain identity, dosage, or formulation testing, and some contain strains without demonstrated relevance to women-specific outcomes [86,94]. Comparative analyses reveal that a considerable proportion of marketed supplements rely on evidence derived from male or mixed cohorts, limiting applicability to women [86,95]. Regulatory frameworks often permit health-adjacent claims without rigorous pre-market validation, contributing to consumer confusion and inconsistent clinical outcomes [96].

Postbiotics have emerged as a promising alternative, offering improved stability, safety, and mechanistic specificity. Experimental and early clinical data suggest potential benefits in fertility, metabolic regulation, and immune modulation; however, women-specific trials remain limited [93,97]. Future research should prioritize strain-resolved clinical trials, standardized outcome measures, and long-term safety assessments, particularly during pregnancy and hormonally dynamic life stages.

### 6.2. Fecal and Vaginal Microbiota Transplantation

Fecal microbiota transplantation (FMT) and vaginal microbiota transplantation (VMT) represent more intensive microbiome-based interventions aimed at restoring ecological balance in severe or refractory dysbiosis. FMT has demonstrated efficacy in select gastrointestinal disorders and is increasingly explored in metabolic and autoimmune conditions that disproportionately affect women [98].

Preclinical and early clinical studies suggest that FMT may modulate systemic inflammation and metabolic dysfunction relevant to polycystic ovary syndrome and autoimmune diseases such as systemic lupus erythematosus [99,100,101]. Animal models demonstrate that transplantation of microbiota from individuals with PCOS can induce metabolic and ovarian dysfunction in germ-free mice, supporting a potential causal role for the gut microbiome in disease pathogenesis [100]. However, human evidence remains limited by small sample sizes and short follow-up durations.

Vaginal microbiota transplantation has emerged as a novel intervention for recurrent bacterial vaginosis unresponsive to standard antibiotic therapy. A landmark proof-of-concept study demonstrated durable restoration of *Lactobacillus*-dominant communities and sustained symptom resolution following VMT [97]. Subsequent studies support biological plausibility, yet large randomized clinical trials are still lacking.

Significant ethical, safety, and regulatory challenges currently limit widespread implementation of both FMT and VMT. These include donor screening, long-term safety monitoring, informed consent, microbiome ownership, and regulatory classification of microbiota-based products [98,99]. Accordingly, these interventions remain experimental. Future efforts should focus on elucidating therapeutic mechanisms, defining optimal donor and recipient characteristics, and developing targeted microbial or metabolite-based alternatives that reduce ethical and regulatory burden.

### 6.3. Personalized Microbiome Medicine for Women

Personalized microbiome medicine represents an emerging frontier in women’s health, enabled by advances in sequencing technologies, computational biology, and artificial intelligence. Rather than applying generalized interventions, precision approaches seek to tailor probiotic selection, dietary modification, and risk prediction based on individual microbial profiles [101,102]. To date, most applications remain exploratory, aiming to generate hypotheses and identify candidate biomarkers rather than inform routine clinical decision-making.

Recent studies have reported associations between vaginal, endometrial, and gut microbiome composition and fertility outcomes, implantation success, and metabolic risk, supporting the conceptual development of microbiome-based risk stratification tools [63,64,65,66,67,68,69,70,71,72,73,74,75,76,77,78,79,80,81,82,83,84,85,86,87,88,89,90,91,92,93,94,95,96,97,98,99,100,101,102,103,104]. Artificial intelligence models that integrate microbiome data with clinical variables have shown promise in retrospective analyses and controlled research settings for predicting reproductive and metabolic outcomes [100,104]. However, these models have largely been developed using limited cohorts and have rarely undergone prospective clinical validation, external replication, or real-world testing.

Despite rapid methodological progress, substantial barriers impede clinical translation. Current studies are frequently constrained by small sample sizes, population homogeneity, lack of standardized analytical pipelines, and restricted access to advanced microbiome testing [63,64,65,66,67,68,69,70,71,72,73,74,75,76,77,78,79,80,81,82,83,84,85,86,87,88,89,90,91,92,93,94,95,96,97,98,99,100,101,102,103]. In addition, inequities in sequencing infrastructure and computational resources raise concerns regarding scalability and implementation across diverse populations. Addressing these challenges will require large, prospective, women-centered cohorts, rigorous external validation of predictive models, and development of cost-effective, clinically interpretable platforms. Until such evidence is available, personalized microbiome medicine should be viewed as a promising but still preclinical approach within women’s health.

## 7. Clinical Commentary: From Bench to Bedside—What Women Need and Research Still Misses

The expanding body of microbiome research reviewed above has generated unprecedented insight into the molecular and ecological determinants of women’s health. However, from a clinical perspective, translation of these discoveries into actionable diagnostic and therapeutic tools remains limited. This bench-to-bedside gap is particularly evident in the care of women with recurrent infections, infertility, pregnancy complications, and hormonally mediated disorders, where microbiome involvement is increasingly suspected but rarely integrated into routine clinical decision-making.

A major barrier to clinical translation is the absence of standardized diagnostic frameworks. Microbiome testing, when available, often relies on specialized external laboratories, involves complex logistics, and generates results that are difficult to interpret within the constraints of routine care. Variability in sampling methods, timing relative to menstrual or reproductive phase, sample handling, and analytical pipelines further limits reproducibility. Critically, reference ranges stratified by age, menstrual cycle phase, pregnancy status, or menopausal state are largely lacking, undermining clinician confidence in incorporating microbiome data into patient management.

Another pressing clinical concern is the widespread and cumulative exposure of women to antibiotics across the lifespan. Broad-spectrum antibiotics are frequently prescribed for recurrent urinary, vaginal, and gastrointestinal symptoms, often without microbiological confirmation. While such treatments may provide short-term symptom relief, they can induce long-lasting disruption of gut and reproductive tract microbial ecosystems. In addition to prescribed antibiotics, chronic low-dose exposure through diet, particularly consumption of animal products produced with antibiotic use, may further compound microbiome perturbations over decades. These patterns underscore the urgent need for antibiotic stewardship strategies that explicitly account for long-term microbiome health in women.

From a translational standpoint, the lack of validated microbial biomarkers represents a critical bottleneck. Although sequencing technologies and multi-omic platforms have advanced rapidly in research settings, clinically actionable signatures capable of distinguishing physiological variation from pathological dysbiosis remain elusive. What is needed are reproducible biomarkers that predict disease risk, guide targeted interventions, and monitor treatment response over time. Without such tools, microbiome-informed care remains aspirational rather than practical.

**Figure 4:**
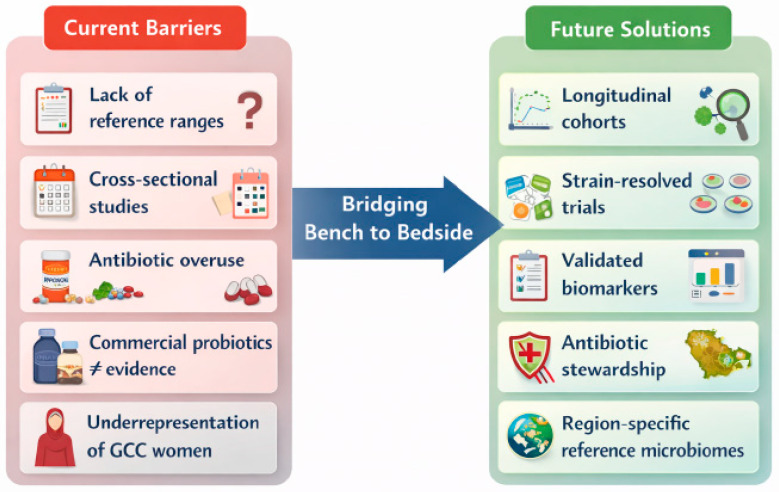
Translational barriers and future priorities for advancing microbiome-informed women’s healthcare. This schematic contrasts current barriers limiting the clinical translation of women’s microbiome research with proposed solutions to bridge discovery from bench to bedside. Key challenges include the absence of life-stage-specific reference ranges, reliance on cross-sectional study designs, widespread antibiotic overuse, discordance between commercial probiotic claims and clinical evidence, and underrepresentation of women from GCC countries. Proposed future directions emphasize longitudinal, women-centered cohorts; strain-resolved and indication-specific clinical trials; development of validated, clinically interpretable microbiome biomarkers; implementation of antibiotic stewardship strategies that account for long-term microbiome health; and establishment of region-specific reference microbiomes to improve generalizability and equity. Together, these priorities outline a roadmap for translating microbiome science into evidence-based, personalized care for women.

Looking forward, several priorities should guide the clinical translation of women’s microbiome research. First, rigorous validation of reproducible, clinically interpretable biomarkers is essential. Second, microbiome-targeted therapies, including probiotics, dietary interventions, and emerging microbiota-based approaches, must be grounded in robust, women-centered clinical trials using standardized protocols. Third, education is foundational: clinicians and patients alike require structured, evidence-based training to interpret microbiome data responsibly and to distinguish validated interventions from commercially driven misinformation. Integrating microbiome science into medical education and continuing professional development will be critical to achieving this goal (Figure 4).

Finally, inclusive research must be prioritized to ensure equitable translation. Women from underrepresented populations, including those in GCC countries, pregnant women, menopausal women, and adolescents, remain disproportionately excluded from longitudinal microbiome studies. Capturing microbiome dynamics across the full female lifespan, rather than isolated snapshots, is essential to understanding how hormonal transitions, lifestyle factors, and environmental exposures shape health outcomes. Addressing these gaps will be key to ensuring that future microbiome-based interventions are both scientifically sound and clinically relevant.

## 8. Conclusions and Future Directions

The evidence synthesized in this review positions the microbiome as a central, multi-organ regulator of women’s health, operating across interconnected biological systems rather than isolated anatomical sites. From the gut and vaginal ecosystems to the skin, breast tissue, and reproductive tract, microbial communities interact dynamically with hormonal fluctuations, immune signaling, metabolic pathways, and environmental exposures throughout the female life course. While advances in sequencing and multi-omic technologies have substantially expanded descriptive knowledge, translation into clinically actionable frameworks remains uneven.

Several consistent gaps emerge across the literature. First, much of the existing evidence is associative, with limited mechanistic or causal validation linking specific microbial taxa, metabolites, or functional pathways to defined clinical outcomes. Second, methodological heterogeneity, including variability in sampling sites, timing relative to hormonal cycles, analytical pipelines, and outcome definitions, continues to limit reproducibility and cross-study comparability. Third, women-specific biological variables such as menstrual cycling, pregnancy, menopause, and contraceptive use are frequently treated as confounders rather than integral determinants of microbiome structure and function. Finally, non-Western populations, including women from GCC countries, remain substantially underrepresented despite distinct dietary patterns, metabolic risk profiles, environmental exposures, and reproductive health challenges.

A unifying theme across gut, vaginal, and reproductive microbiome research is the presence of tightly coupled microbiome–endocrine–immune axes. The gut microbiome influences systemic estrogen availability through the estrobolome, modulates metabolic and inflammatory tone, and communicates bidirectionally with the brain via neuroendocrine pathways. In parallel, the vaginal microbiome directly shapes local immune defenses, epithelial integrity, and susceptibility to infection, infertility, and adverse pregnancy outcomes. Increasing evidence suggests that these systems are interconnected rather than independent: gut-derived metabolites and immune mediators may influence reproductive tract microbial ecosystems, while local dysbiosis may reciprocally affect systemic inflammation and endocrine regulation. Conceptualizing women’s health through an integrated gut–vaginal–endocrine framework provides a biologically coherent lens through which diverse conditions, ranging from infertility and recurrent infections to mood disorders, metabolic disease, and hormone-sensitive cancers, can be understood as interconnected manifestations of systemic dysregulation.

Future research must therefore move beyond compartmentalized, single-site analyses toward longitudinal, systems-level study designs spanning multiple body sites and life stages. Priority should be given to integrative approaches combining microbial taxonomy with functional readouts such as metabolomics, transcriptomics, and immune profiling to identify causal mechanisms and therapeutic targets. Clinical translation will depend on the development of standardized, validated biomarkers capable of guiding personalized interventions. Importantly, microbiome-based therapies—whether probiotics, postbiotics, or microbiota transplantation—must be evaluated in strain-resolved, women-centered clinical trials rather than extrapolated from male-dominated or mixed cohorts.

From a regional perspective, the UAE and broader GCC represent a critical and underexplored context for women’s microbiome research. High prevalence of metabolic disease, extensive antibiotic exposure, distinctive dietary and lifestyle patterns, and rapid healthcare modernization create both challenges and opportunities. Establishing region-specific reference microbiomes, longitudinal women’s health cohorts, and culturally tailored intervention studies will be essential to ensure equitable translation of microbiome science. Failure to account for regional biological and environmental differences, including those prevalent in GCC populations, risks limiting the accuracy, reproducibility, and translational impact of women’s microbiome research. Establishing region-specific reference microbiomes and longitudinal cohorts in the GCC is therefore essential for advancing globally applicable precision microbiome medicine

In conclusion, the women’s microbiome represents a powerful but underutilized axis in precision medicine. Realizing its full potential will require conceptual integration across biological systems, rigorous methodological standardization, inclusive population representation, and sustained collaboration between basic scientists, clinicians, and health systems. By aligning molecular discovery with clinical reality and global diversity, microbiome research has the potential to meaningfully advance personalized, evidence-based care for women across the lifespan.

## Figures and Tables

**Table 1:** Key microbiome sites, mechanisms, and clinical relevance in women’s health.

*Microbiome Site*	*Molecular Mechanism*	*Key Metabolites/Enzymes*	*Clinical Relevance*
*Gut*	Estrobolome activity	β-glucuronidase	Estrogen recycling, cancer risk
*Vagina*	Acidification	Lactic acid	BV, STI prevention
*Gut–brain*	Neuroimmune signaling	SCFAs, indoles	PMS, postpartum depression
*Breast*	Genotoxic stress	Colibactin	Cancer progression

## Data Availability

No new data were created or analyzed in this study. Data sharing is not applicable to this article.
